# SpecEncoder: deep metric learning for accurate peptide identification in proteomics

**DOI:** 10.1093/bioinformatics/btae220

**Published:** 2024-06-28

**Authors:** Kaiyuan Liu, Chenghua Tao, Yuzhen Ye, Haixu Tang

**Affiliations:** Department of Computer Science, Luddy School of Informatics, Computing and Engineering, Indiana University, IN 47408, United States; Department of Computer Science, Luddy School of Informatics, Computing and Engineering, Indiana University, IN 47408, United States; Department of Computer Science, Luddy School of Informatics, Computing and Engineering, Indiana University, IN 47408, United States; Department of Computer Science, Luddy School of Informatics, Computing and Engineering, Indiana University, IN 47408, United States

## Abstract

**Motivation:**

Tandem mass spectrometry (MS/MS) is a crucial technology for large-scale proteomic analysis. The protein database search or the spectral library search are commonly used for peptide identification from MS/MS spectra, which, however, may face challenges due to experimental variations between replicated spectra and similar fragmentation patterns among distinct peptides. To address this challenge, we present SpecEncoder, a deep metric learning approach to address these challenges by transforming MS/MS spectra into robust and sensitive embedding vectors in a latent space. The SpecEncoder model can also embed predicted MS/MS spectra of peptides, enabling a hybrid search approach that combines spectral library and protein database searches for peptide identification.

**Results:**

We evaluated SpecEncoder on three large human proteomics datasets, and the results showed a consistent improvement in peptide identification. For spectral library search, SpecEncoder identifies 1%–2% more unique peptides (and PSMs) than SpectraST. For protein database search, it identifies 6%–15% more unique peptides than MSGF+ enhanced by Percolator, Furthermore, SpecEncoder identified 6%–12% additional unique peptides when utilizing a combined library of experimental and predicted spectra. SpecEncoder can also identify more peptides when compared to deep-learning enhanced methods (MSFragger boosted by MSBooster). These results demonstrate SpecEncoder’s potential to enhance peptide identification for proteomic data analyses.

**Availability and Implementation:**

The source code and scripts for SpecEncoder and peptide identification are available on GitHub at https://github.com/lkytal/SpecEncoder. Contact: hatang@iu.edu.

## 1 Introduction

Tandem mass spectrometry (MS/MS) using Collision-Induced Dissociation or Higher-energy Collisional Dissociation (HCD) has become an indispensable technique in proteomics for identifying and quantifying proteins and their post-translational modifications (PTMs) in complex biological samples (Aebersold and Mann 2003). In a typical bottom-up proteomics workflow, proteins are first digested into peptides by trypsin, and subsequently separated and analyzed via liquid chromatography coupled tandem mass spectrometry (LC-MS/MS) (Zhang *et al.* 2013). As a result, the development of computational methods for proteomic data analyses, in particular for the identification of peptides and/or their PTMs with high confidence, has become a fundamental research problem in proteomics and has been extensively studied over the past two decades, resulting in hundreds of peptide identification algorithms (Eng *et al.* 2011).

The early efforts in peptide identification were focused on database-searching strategies. The algorithms such as SEQUEST (Eng *et al.* 1994), Mascot (Hirosawa *et al.* 1993), X! Tandem (Craig and Beavis 2003), and OMSSA (Geer *et al.* 2004) compare experimental MS/MS spectra against the tryptic peptides in a target protein database and compute an empirical score for each putative peptide-spectrum match (PSM) that measures the matching of desirable fragmentation pattern in the experimental spectra. More recent database search engines use more sophisticated scoring functions to improve the sensitivity of peptide identification (Cox *et al.* 2011, Kim and Pevzner 2014).

In addition to the database-searching algorithms, spectral library searching emerged as a complementary approach (Yates *et al.* 1998). It aims to search experimental spectra against a reference spectral library consisting of previously identified MS/MS spectra with annotated peptides. Data repositories like MassIVE, PRIDE, and ProteomeXchange (Vizcaíno *et al.* 2014) have enabled the broad dissemination of proteomics datasets across the research community. As a result, large spectral libraries were built from the MS/MS spectra curated from these datasets. On the other hand, the development of software tools like SpectraST (Lam *et al.* 2007) allows for the fast spectral library searching of massive proteomics data (Lam *et al.* 2008, Frank *et al.* 2011, Deutsch *et al.* 2018).

For both approaches, the empirical scoring schemes used by different methods are crucial for their performance, as they often encounter two primary challenges. First, peptides with slight sequence variances may display comparable fragmentation patterns, thereby raising the risk of false peptide identification. In addition, replicated spectra of identical peptides can show considerable distinctions due to experimental variations, leading to diminished confidence in accurate peptide identifications.

Many scoring functions have been proposed to evaluate the quality of peptide identifications. For database search approaches, early tools like SEQUEST used relatively simple models that summed peak intensities of matched fragment ions. Later algorithms incorporate more sophisticated statistical models to better distinguish between true and false PSMs. For example, Andromeda (Cox *et al.* 2011) uses a naive Bayes classifier to calculate joint PSM probabilities; MSGF+ (Kim and Pevzner 2014) combines several scoring functions including precursor mass accuracy and fragment ion intensity distributions; Percolator (Käll *et al.* 2007) trains SVM classifiers to refine existing scores. For spectral library search approaches, similarity scoring functions are used to evaluate spectrum-spectrum matches (SSMs), e.g. SpectraST uses cosine similarity based on ranked intensities instead of raw intensities.

More recently, deep-learning approaches have been proposed to learn a latent representation of MS/MS spectra so that the similarity between two spectra can then be calculated by comparing their representations (Huber *et al.* 2021, Guo *et al.* 2023). For example, GLEAMS (Bittremieux *et al.* 2022) embeds spectra into latent vectors for large-scale spectrum clustering; DLEAMSE (Qin *et al.* 2021) uses the Siamese Network for spectral similarity scoring, and MSBooster (Yang *et al.* 2023) uses deep-learning models to improve peptide identifications reported by the peptide search engine MSFragger (Kong *et al.* 2017).

Despite these methods, we believe a more informative and discriminative representation of MS/MS spectra can further improve peptide identification. In this paper, we propose a deep metric learning approach called *SpecEncoder*, to learn a latent representation of peptide MS/MS spectra such that the spectra of different peptides are more distinguishable in the latent space. Also, the model can be used to perform protein database searching by producing latent vectors from predicted full MS/MS spectra (e.g. by using PredFull; Liu *et al.* 2020a). Furthermore, a hybrid-searching strategy that combines the spectral library and the peptide database searching becomes possible as the model can embed peptides into the same latent space.

## 2 Methods

### 2.1 SpecEncoder model architecture

**Figure 1.**
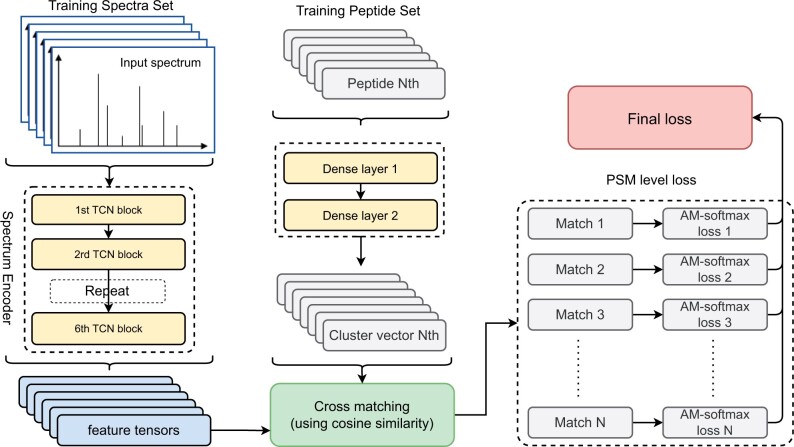
The architecture of the SpecEncoder model. The final loss is the sum of all matches.

As depicted in Fig. 1, the model comprises two components: a *spectrum encoder* transforming an input MS/MS into a fixed-size latent vector, and a cross-matching component that computes the loss function based on the comparison of the vectors transformed from matching spectra and peptides.

In the spectrum encoder, any input MS/MS spectrum is first transformed into a vector representation in 20 000 dimensions (using m/z bin of 0.1) and then extended to 20 480 through zero-padding, following the procedure described in PredFull (Liu *et al.* 2020a). These vectors, along with their reversed vectors, form an input matrix to the spectrum encoder. The spectrum encoder consists of six consecutive Temporal Convolutional Network (TCN) blocks (Bai *et al.* 2019) and down-sampling layers, used to capture the relationship between the observed peaks in the input spectrum. As demonstrated in its original publications (Bai *et al.* 2019), the TCN layer has proven to be well-suited for tasks involving sequence data, making them an effective choice for processing spectral data (Liu *et al.* 2023). By this design, the lower TCN blocks have larger receptive fields whereas the deeper TCN blocks have smaller receptive fields. Each TCN block will have halved receptive fields but with approximately 1.5 times more channels compared to the block in front of it. The meta-information of the spectra such as the charge, the precursor mass, and the normalized collision energy, when available, is incorporated as additional input to the spectrum encoder, which is transformed by a linear layer (with the Sigmoid activation function) into a vector concatenated to the end of the tensor output by the last TCN block. Finally, an additional dense layer further transforms the output into the latent feature vector in 256 dimensions (Fig. 1). Peptides are encoded using a one-hot representation with a length limitation of up to 30 amino acid residues, as most peptides in proteomic studies are 30 or fewer amino acids long. However, this length constraint can be alleviated to accommodate any desired peptide length. Peptides shorter than 30 amino acids are padded with zeros to maintain a fixed length of 30. Then, two densely connected layers will transform the encoded peptide sequence into a latent vector of the same size (256 dimensions).

The cross-matching component in the SpecEncoder will compute the *additive margin softmax (am-softmax)* (Wang *et al.* 2018a) loss between the latent vectors converted from matching spectrum and peptide. The am-softmax loss function is a modification of the traditional softmax loss that adds a margin to the target logit. The addition of margin encourages the model to learn discriminative features while reducing the intra-class variance, which is crucial for our purpose to distinguish the spectra from different peptides in the peptide identification results. The AM-softmax loss has been demonstrated to enhance the discriminative power of deep metric learning models, as shown in facial recognition tasks in the original publication (Wang *et al.* 2018a).

During the training process, SpecEncoder treats each unique peptide and its experimental MS/MS spectra in the training dataset as a separate class. The goal is to match the latent vector of a peptide with the latent vectors of the experimental spectra resulting from the same peptide, such that they have the highest cosine similarity. Conversely, the similarities between the latent vectors of unmatched peptides and spectra from different classes are relatively low. In addition to the main classification task, numerous auxiliary tasks were performed to stabilize the training process. These included reconstruction of the input spectra using a decoder, ensuring that no critical information in the input spectrum is lost through the spectrum encoder.

We note that the cross-matching component is large and complex, containing 160 million parameters, while the spectrum encoder is relatively small, with around 3.7 million parameters. By this design, the large cross-matching component was only utilized to guide the encoder to learn a good representation during training, while after training, only the small encoder network is needed for subsequent peptide identification.

### 2.2 Peptide identification using SpecEncoder

**Figure 2.**
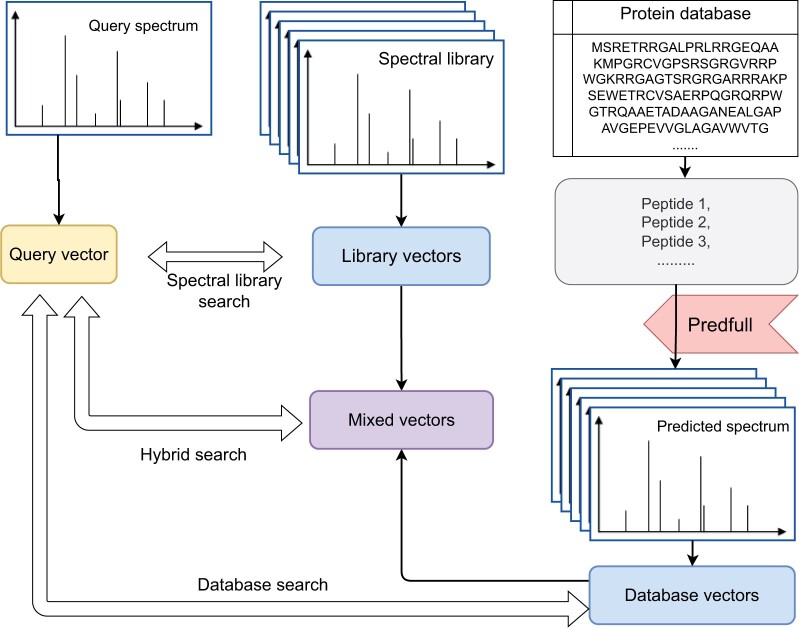
The workflow for the hybrid searching of MS/MS spectra against the combined peptide spectral library and the protein database by using SpecEncoder. The spectra in the spectral library are first embedded into the set of *library vectors*; for the remaining peptides in the target protein database that do not have their known experimental spectra in the spectral library, PredFull will be exploited to predict their spectra, which are subsequently embedded into the set of *database vectors*. Finally, the library and database vectors will be merged into the *mixed vector library* that is subjected to the hybrid searching of the query spectra in an input proteomics dataset. The workflow can also simplified for the spectral library searching or the protein database search, where only the library vectors or the database vectors are used as the target, respectively.

The goal of this study is to demonstrate that SpecEncoder can improve peptide identifications through its latent representations that can better distinguish the spectra of the same peptide from those of different peptides. In fact, the latent feature vectors of MS/MS spectra represented by SpecEncoder (more specifically, the spectrum encoder trained in the SpecEncoder model) can be exploited for different applications, as illustrated in Fig. 2.

First, SpecEncoder can be employed for spectral library searching: instead of searching an experimental MS/MS spectra against the spectra in the library (e.g. by using SpectraST; Lam *et al.* 2007), one may compare its latent vector against pre-computed latent vectors of all the spectra in the library. Here, we expect the cosine similarities between the latent vectors would exhibit greater discriminating power than the similarity between the MS/MS spectra that is often adopted as the metric to rank the hits in the spectral library searching. The deep metric learning approach adopted by SpecEncoder can be viewed as a deep-learning algorithm to automatically learn a non-linear transformation function to transform spectra into latent vectors such that the cosine similarity in the latent space is equivalent to a complex non-linear scoring function for spectra.

Next, SpecEncoder can also be exploited for protein database searching, where the (tryptic) peptides instead of spectra are transformed into the latent vectors subjected to the searching of input experimental MS/MS spectra. Specifically, we will first perform an *in silico* digestion (e.g. using trypsin) of the proteins in the target protein database and then predict the MS/MS spectra of all resulting peptides using PredFull (Liu *et al.* 2020a). Afterward, we use SpecEncoder to further convert the predicted spectra into the latent vectors, which can then be searched against in the same manner as in the spectral library searching. Similarly, comparison of the latent vectors is equivalent to scoring PSMs using a complex non-linear scoring function.

Lastly, SpecEncoder allows for a hybrid search against both spectral library and protein database. Specifically, we can construct a *mixed vector library* by merging the latent vectors transformed from the experimental MS/MS spectra in the library (for the peptides in the protein database whose spectra have been collected in the library) and those from the predicted spectra (for the peptides whose spectra have not been collected). We note that although this approach seems straightforward, it is not feasible without the embedding method presented by SpecEncoder, as the conventional methods for spectral library searching and protein database searching use completely different scoring functions, and thus their results cannot be directly compared and ranked. Furthermore, the score distributions of SSMs and the PSMs are different and thus the false discovery rate (FDRs) cannot be estimated using the target-decoy searching (TDS) approach. In contrast, the SpecEncoder model transforms both experimental and predicted spectra into vectors in the same latent space. As a result, regardless of the source of the spectra, the vector-vector matches (VVMs) follow the same score distribution, allowing for direct estimation of FDRs in VVMs using a TDS approach (see below). This approach can be further generalized into a progressively updated manner, where the latent vectors of experimental spectra are gradually added into the mixed vector library when they become available, replacing the vectors concerted from predicted spectra on the same peptides.

### 2.3 Implementation and training

We implemented the SpecEncoder model using Keras and TensorFlow. We used the RAdam optimizer (Liu *et al.* 2020b) with a learning rate of 0.0072 to train the model for 150 epochs. The complete training process takes around 12 h using eight cards of NVIDIA A6000 GPU. We picked the model weight from the epoch that yields the best accuracy on the cross-validation dataset after the training is completed. Separate models were trained for charge 2+ and charge 3+ spectra, as we found that training individual models for each charge performed slightly better. This may be due to (i) spectra of different charges do not share significant common information, (ii) training separate models reduces the possibility of confusion and accelerates the convergence of the models, and (iii) the numbers of training data of different charge states are very imbalanced. Since we know the charge of each query spectrum, using separate models does not pose a limitation. To enhance the model’s robustness and ability to handle experimental noises in the input spectra, we intentionally added Gaussian noise into the input spectra during training, and observed slightly improved performance. The encoder part of SpecEncoder can transform around 10 000 input spectra into latent vectors in around 11 s using a single NVIDIA A6000.

The SpecEncoder model was trained on a large dataset of HCD spectra and their annotated peptides, derived from multiple spectral libraries and experimental datasets, including the NIST HCD library (Yang *et al.* 2017), the NIST Synthetic HCD library (Yang *et al.* 2017), the Human HCD library from MassIVE (Wang *et al.* 2018b), and the synthetic HCD library from ProteomeTools (Zolg *et al.* 2017). In total, we collected approximately 1.5 million charge 2+ spectra from 70 000 unique peptides, and around 1 million charge 3+ spectra from 50 000 unique peptides. To ensure the robustness and diversity of the training data while avoiding redundancy, we retained a maximum of 10 replicates for each unique peptide. For each unique peptide, one spectrum was randomly selected for cross-validation while all other spectra were used for training. Clearly, if a unique peptide has no replicates we did not use it for either purpose. Finally, for charge 2+ spectra, 1 006 797 spectra were selected for training and 547 148 spectra were used for cross-validation. Whereas for charge 3+ spectra, 694 273 spectra were selected for training and 401 830 spectra were used for cross-validation. It is important to note that the model can be easily extended to train on spectra of other charges, such as charge 1+ and charge 4+, provided sufficient training spectra are available. However, for the purpose of demonstrating the model’s utility, we focused on the 2+ and 3+ spectra in the paper.

### 2.4 Evaluation of SpecEncoder for peptide identification in proteomics

Three independent large-scale human proteomics datasets (MassIVE IDs: MSV000079526 (Wilhelm *et al.* 2014), MSV000081563 (Bekker-Jensen *et al.* 2017) and MSV000079514 (Pinto *et al.* 2014)) were used to evaluate the performance of SpecEncoder. Each dataset contains over one thousand RAW files, and tens of millions of MS/MS spectra, thus suitable to provide a fair and comprehensive test. For each dataset, all spectra in the raw files are used, we first convert RAW files to the MGF format using ProteoWizard/msConvert (Kessner *et al.* 2008) for subsequent peptide identification. Currently, the SpecEncoder model is trained on data-dependent acquisition spectra only, and will explore extending SpecEncoder to data-independent acquisition spectra in future works. Table 1 summarizes the three datasets.

**Table 1.** Summary of the spectra dataset used for testing.

ID	Description	Number of raw files	Number of MS/MS spectra
MSV000079526	36 Human tissues and body fluids	1055	13 270 627
MSV000081563	HeLa proteome of ∼584K unique	1021	22 284 971
	peptide and ∼14 200 protein isoforms		
MSV000079514	30 Histologically human samples of	2207	24 939 983
	17 adult tissues, 7 fetal tissues		
	6 purified primary hematopoietic cells		

We evaluated SpecEncoder for each of the three searching approaches described above. We first compared SpecEncoder with SpectraST, one of the best-performing spectral library searching tools for peptide identification, on the three human proteomics datasets against the NIST spectral library consisting of 413 729 MS/MS spectra from 283 006 human peptides (Consortium 2015). Next, for the protein database-searching approach, we compared SpecEncoder (and SpecEncoder-based hybrid searching) with MSGF+ (Kim and Pevzner 2014) enhanced by the post-processing tool Percolator (Käll *et al.* 2007), which is considered one of the standard peptide identification protocols in proteomics, on the same three human proteomics datasets against the UniProt human protein database. In all of these scenarios, we used the TDS approach to estimate the FDR of the identification results.

These testing datasets surely contain spectra that yield form peptides already seen in the training datasets, however, as we evaluate performances on independent datasets, SpecEncoder must be able to learn transferable latent features rather than solely memorizing instances from the training data, as replicate spectra from different datasets can exhibit significant variations. Moreover, within the three independent datasets used for evaluation, more than 60% of the identified peptides were never presented in the training data. Furthermore, recognizing replicate spectra is part of the goal in PSM identification; especially for spectral library search, where all identified spectra exist in the search spectral library.

When running SpectraST, we set a precursor ion tolerance of 0.05 Da, and the “-sR” flag was used to specify the need for re-calibrating precursor masses. For MSGF+, we selected trypsin as the enzyme for peptide digestion, with a specified minimum peptide length of six amino acids and a maximum length of 30. We allowed for up to two missed cleavages and considered peptides with a charge state ranging from a minimum of 1+ to a maximum of 4+. Additionally, we adopted a precursor ion tolerance of 0.05 Da for both MS1 and MS2 spectra. We also used Percolator (Käll *et al.* 2007) to enhance the MSGF+ results. When running Percolator, we specified ‘rankdelta’ to be 1 and enabled the ‘newDat’ flag to write a new file that outputs Percolator’s posterior error probabilities.

### 2.5 Data and code availability

The proteomic data used for this study were taken from the MassIVE proteomics data repository, with the identifiers MSV000079526, MSV000081563, and MSV000079514, respectively. The source code and scripts for SpecEncoder and peptide identification are available on GitHub at https://github.com/lkytal/SpecEncoder.

## 3. Results

### 3.1 Measuring similarities between MS/MS spectra in the embedded latent space

**Figure 3.**
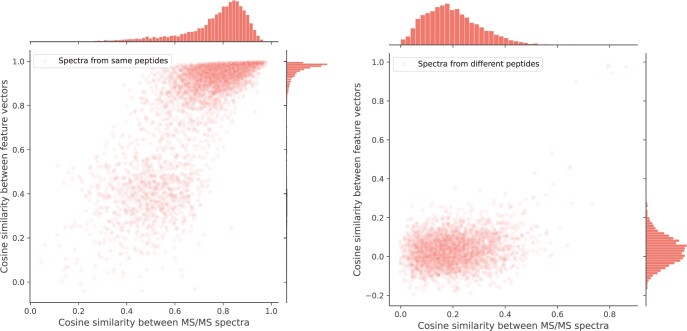
The cosine similarities between pairs of MS/MS spectra (*x*-axis) versus the cosine similarities of their derived latent vectors (*y*-axis), for the spectra of the same peptides (left) and those of different peptides (right), respectively. Apparently, for the spectra of the same peptides, their derived latent vectors are almost identical (with cosine similarities close to 1), even for those spectra sharing low similarities. In contrast, the similarities between the latent vectors derived from the spectra of different peptides are relatively lower, although they are also lifted to a higher level.

We first show the effectiveness of the SpecEncoder embedding in distinguishing the spectra from different peptides in Fig. 3. The left panel of the figure compares the cosine similarities between the replicated experimental spectra of the same peptides (*x*-axis) versus the cosine similarities between their latent vectors (*y*-axis), whereas the right panel shows the same thing but for the spectra from different peptides. The results indicate that for the spectra of the same peptides, their latent vectors are highly similar (with similarities close to 1), even for those spectra with relatively low similarities (e.g. <0.5). On the other hand, for the spectra of different peptides, their similarities in the latent space remain low. These results suggest that the spectrum embedding by SpecEncoder amplifies the gap between the similarities of the spectra from the same peptides and those of spectra from different peptides.

As a result, the comparison of spectra in the latent space leads to better separation of the true matches between experimental MS/MS spectra (i.e. those of the same peptides) and the false matches (those of different peptides) in the database or library searching, and consequently, more true identification results can be retained under the same FDR cutoff, as shown below.

### 3.2 Peptide identification by spectral library searching

Next, we evaluated SpecEncoder for spectral library searching in comparison with SpectraST (Lam *et al.* 2006), which is a widely adopted and proven tool for peptide identification. The NIST spectral library was used as the target library, and three human proteomic datasets were used in this experiment (see Section 2). For running SpectraST, we converted the NIST library into the database format used by its associated tools and generated the decoy spectral library using the tool provided by SpectraST. For SpecEncoder-based library searching, we transformed the spectra in both the target (i.e. the NIST library) and the decoy library (generated by SpectraST) into the target and decoy latent vectors, respectively. The combined library of the target/decoy vectors is then searched against the latent vectors transformed by SpecEncoder from the query spectra in the human proteomics datasets.

For each candidate spectrum in the combined target/decoy library with matched charge and precursor mass (within 10 ppm difference), we computed the cosine similarity between its latent vector and the query vector. The VVM with the highest similarity for each query vector is considered as the putative identification (i.e. the top hit), and the peptide corresponding to the matched spectrum is reported as the identified peptide, no matter whether it comes from the target or the decoy library. Finally, for both SpectraST and SpecEncoder, we estimated the FDR following the standard TDS strategy, and the similarity cutoff is selected based on FDR of 0.01. Note that we perform the FDR control on peptide level, while the results using spectrum level FDR only exhibit slightly different.

**Figure 4.**
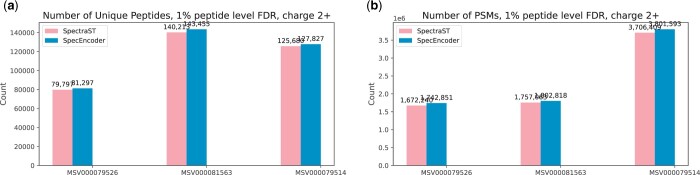
Numbers of spectra (PSMs) and unique peptides identified by SpecEncoder and SpectraST on the charge 2+ spectra in three human proteomics datasets at peptide level FDR of 0.01.

**Figure 5.**
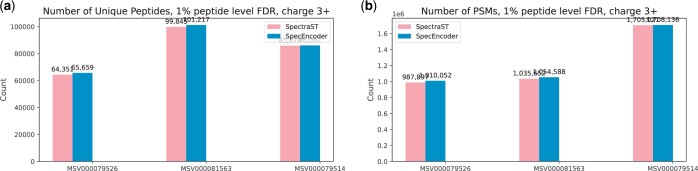
Numbers of spectra (PSMs) and unique peptides identified by SpecEncoder and SpectraST on the charge 3+ spectra in three human proteomics datasets at peptide level FDR of 0.01.

Figures 4 and 5 show the results of SpectraST and SpecEncoder on each dataset, including the total number of identified MS/MS spectra and unique peptides. For both 2+ and 3+ spectra across all three datasets, SpecEncoder consistently outperforms SpectraST, identifying 1%–2% more MS/MS spectra and 1% more unique peptides on the 2+ spectra (Fig. 4), and approximately 1% more MS/MS spectra and 1%–2% more unique peptides on the 3+ spectra (Fig. 5). The performance improvement by SpecEncoder is slightly smaller on the 3+ spectra than on the 2+ spectra, perhaps because of the relatively smaller training data used for the embedding of the 3+ spectra compared to the 2+ spectra, and the 3+ spectra are inherently more complex.

### 3.3 Peptide identification by protein database searching and hybrid searching

Although the spectral library searching could yield decent peptide identification, it suffers from the incompleteness of the target spectra library. For instance, the NIST spectral library contains only about 30% tryptic peptides in all human proteins. As a result, peptide identification in proteomic studies is commonly conducted by using protein database search engines that directly compare the experimental MS/MS spectra and peptide sequences in a target database based on empirical scoring functions. To elucidate that the latent representation could also be used to improve peptide identification in protein database searching, we conducted a comparative analysis between SpecEncoder and the database search engine MSGF+ (Kim and Pevzner 2014), which is renowned for its ability to handle spectra of various types and is widely used in proteomics research due to its high sensitivity and accuracy. The benchmarking experiments (Kim and Pevzner 2014) showed that MSGF+ can exhibit comparable or superior performance to other popular peptide identification tools. Consequently, we choose to only compare SpecEncoder against MSGF+ and expect the results would also apply to other peptide identification tools.

In this experiment, we used the UniProt human proteins and performed *in silico* trypsin digestion to derive the target peptides. By allowing for up to two missed cleavages, around 2.4 million human peptides in length between 6 and 30 amino acids were retained to form the target database. We used MSGF+ to create a decoy database for its own search. For SpecEncoder, we first predicted the MS/MS spectra of all tryptic peptides using PredFull (Liu *et al.* 2020a), and then transformed the predicted spectra into the latent vectors by using SpecEncoder. The mixed library of latent vectors occupies approximately 4.6 GB of space. Similar to the spectral library searching, for each query experimental spectrum, we consider the candidate target peptides with the matched charge and precursor mass (within 10 ppm), and among them, select the peptide whose predicted spectrum receives the highest cosine similarity in the latent space with the query. For all experiments, we assumed a fixed carbamidomethyl modification on cysteine residues, while no other modifications were considered. However, SpecEncoder can be easily extended to support common PTMs.

**Figure 6.**
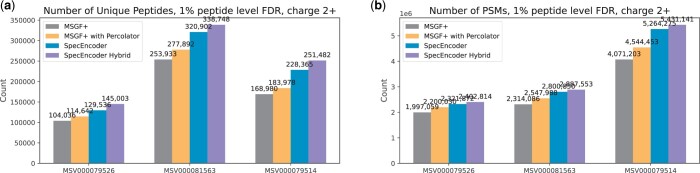
For charge 2+ spectra, the numbers of spectra (PSMs) and unique peptides identified by MSGF+ and SpecEncoder on the 2+ spectra in the three human proteomics datasets, respectively. The 1% FDR cutoff at the peptide level (calculated on each raw file) is applied.

We compared the peptide identification results of SpecEncoder with the results directly from MSGF+, and the results from MSGF+ enhanced by Percolator (Käll *et al.* 2007), a post-processing tool that uses semi-supervised learning to improve the scoring of PSMs. As depicted in Fig. 6, SpecEncoder using predicted spectra yields superior performance compared to both MSGF+ and MSGF+/Percolator. When compared to the results from MSGF+ enhanced by Percolator, SpecEncoder identified around 5%–15% more spectra and unique peptides under the same peptide level FDR cutoff (0.01) from the 2+ spectra (Fig. 6). We note that MSGF+/Percolator needs two steps of data processing (by MSGF+ and by Percolator), whereas SpecEncoder needs only one step of spectra searching after the latent vectors are derived from the peptides in the protein database. This makes SpecEncoder potentially more efficient for processing large-scale proteomic datasets.

**Figure 7.**
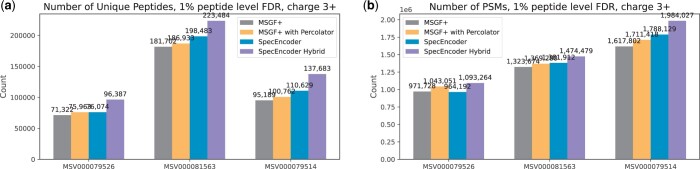
For charge 3+ spectra, the numbers of spectra (PSMs) and unique peptides identified by SpecEncoder and MSGF+ on the 2+ spectra in three human proteomics datasets, respectively. A 1% FDR cutoff at the peptide level (calculated on each raw file) is applied.

SpecEncoder also outperforms raw MSGF+ on the 3+ spectra. Although the complex fragmentation pattern of the 3+ spectra may impose a greater challenge for learning an optimal metric to distinguish these spectra, SpecEncoder can still achieve better performance (Fig. 7).

It is worth noting that the direct comparison between the experimental and predicted spectra (e.g. using cosine similarity) could not yield superior performance, as the predicted spectra are not sufficiently similar to the experimental spectra. In fact, direct spectral searching using predicted spectra yields slightly worse results compared to the protein database searching using MSGF+/Percolator, indicating the cosine similarity measure between predicted and experimental spectra is not necessarily better than the empirical scoring function used by MSGF+/Percolator to rank the PSMs. On the other hand, by transforming both the query spectra and the predicted spectra into latent vectors, SpecEncoder achieved better peptide identification than MSGF+/Percolator.

Finally, we evaluated the performance of the hybrid-searching approach using SpecEncoder. Here, we constructed a mixed vector library by combining the vectors converted from the experimental spectra of human peptides in the NIST spectral library and the vectors from the predicted spectra of the tryptic peptides whose experimental spectra are not collected in the NIST library. As depicted in Fig. 6, the hybrid searching identified additional 4%–12% spectra and unique peptides on 2+ spectra across all three datasets. Similar results can also be observed on 3+ spectra (Fig. 7). These results show that the enhancement of SpecEncoder for peptide identification can be achieved on both the experimental and predicted spectra, allowing it to be used for the spectra searching against the merged library of latent vectors of both types of spectra.

Additionally, to provide insight into how SpecEncoder’s performance compared to deep-learning-based methods, we conducted a comparative analysis between SpecEncoder and the deep-learning-based peptide identification method, MSFragger combined with MSBooster (Yang *et al.* 2023), utilizing the dataset MSV000079526 as a benchmark. Here, we run MSFragger via FragPipe GUI, with the default configuration and MSBooster (with Percolator, which is required by MSBooster) enabled within the “validation” section. As shown in Table 2, even though MSFragger/MSBooster identified more PSMs, SpecEncoder Hybrid identified significantly more unique peptides than MSFragger/MSBooster on both 2+ and 3+ charged spectra. As SpecEncoder and MSBooster employ different deep-learning strategies and training data, this could lead to varying performances on different charge states and spectral patterns.

**Table 2.** The numbers of spectra (PSMs) and unique peptides identified by SpecEncoder compared to MSFragger with MSBooster on human proteomics dataset MSV000079526.

	Number of unique peptides		Number of PSMs	
	Charge 2+	Charge 3+	Charge 2+	Charge 3+
SpecEncoder Hybrid	145 003	96 387	2 402 814	1 093 264
MSFragger + MSBooster	110 188	81 579	2 464 826	1 332 467

### 3.4 Ablation study on network architecture

An ablation study was conducted to investigate the influence of the network size and the embedding dimension on model performance. For simplification, we only show the accuracy on the validation set, while we expect the performance on the independent datasets will follow the same trend. Concerning the network size, our findings demonstrate that the current scale of the model is sufficiently large to achieve good performance, while further increasing the network size would incur significantly higher computational costs with diminishing returns in accuracy gains (Table 3). We also analyzed the performance fluctuations across different embedding dimensions. As shown in Table 3, halving the dimension to 128 results in a significant drop in performance, with even smaller dimensions performing worse. On the other hand, increasing the dimension to 512 yields only minor improvements in accuracy. Based on these findings, we chose an embedding dimension of 256 as a balanced configuration that provides robust performance without excessive computational overhead.

**Table 3.** Ablation study of accuracy on the validation set.

**Accuracy**	**0.885**	**0.959**	**0.982**	**0.990**
Vector dimension	64	128	256	512
Accuracy	0.617	0.891	0.982	0.984

## 4. Conclusion

In this paper, we present SpecEncoder, a novel metric learning model for peptide identification in mass spectrometry-based proteomics. SpecEncoder addresses the limitations of traditional methods that rely on cosine similarity or other metrics to measure spectral similarity by exploiting a deep-learning approach to learn a metric that measures the similarity between mass spectra in an embedded latent space. The model structure of SpecEncoder is based on PepNet, a deep neural network architecture that utilizes sequential TCN blocks to capture long-range dependencies in MS/MS spectra effectively.

Our results demonstrate the versatility of SpecEncoder’s latent vector representation for multiple peptide identification scenarios, including spectral library searching, protein database searching, and hybrid searching combining both. SpecEncoder outperforms SpectraST by identifying around 1%–2% more unique peptides on the charge 2+ spectra while showing a comparable performance for charge 3+ spectra. When comparing the database-searching tool MSGF+ with the enhancement of a post-processing tool Percolator, SpecEncoder identifies around 6%–15% more spectra and unique peptides under the same FDR cutoff (0.01). Additionally, the SpecEncoder-based hybrid-searching method gains an additional 6%–12% more identification results.

We anticipate that SpecEncoder’s performance can be further improved with more training data. In this study, we only performed database searches on charge 2+ and charge 3+ spectra. However, this approach can be easily extended to the spectra of other charges (e.g. 1+ and 4+). This flexibility will facilitate its use for peptide identification in a broad range of applications. Our future work will focus on enhancing the model’s performance and extending it to other charges and types of MS/MS spectra.
